# Recurrent testicular torsion post orchidopexy ‐ an occult emergency: a systematic review

**DOI:** 10.1111/ans.17592

**Published:** 2022-03-07

**Authors:** Mikayla van Welie, Liang G. Qu, Ahmed Adam, Nathan Lawrentschuk, Abdullah E. Laher

**Affiliations:** ^1^ Department of Emergency Medicine Faculty of Health Sciences, University of the Witwatersrand Johannesburg South Africa; ^2^ Department of Urology Olivia Newton John Cancer Research Institute, Austin Health Heidelberg Victoria Australia; ^3^ Division of Urology, Faculty of Health Sciences University of the Witwatersrand Johannesburg South Africa; ^4^ Department of Urology Royal Melbourne Hospital Melbourne Victoria Australia

**Keywords:** acute scrotum, RTT, recurrence, recurrent, spermatic cord torsion, testicular torsion, testicular torsion, testis torsion, TT

## Abstract

**Background:**

Recurrent Testicular Torsion (RTT) is a rarely reported event after previous testicular torsion (TT) repair. Both conditions have similar signs and symptoms. Various techniques have been attempted to reduce the incidence of retorsion. This review assesses the presentation, diagnosis, risk factors, management and outcomes associated with RTT.

**Methods:**

After PROSPERO Registration (CRD42021258997), a systematic search of PubMed, Google Scholar, Embase, Scopus, Web of Science, Cochrane Database of Systematic Reviews, Global Index Medicus and Cumulative Index to Nursing and Allied Health Literature (CIANHL) was performed using specific search terms. Study metadata including patient demographics, orchidopexy techniques, RTT rates and RTT timing were extracted.

**Results:**

Twenty‐six articles, comprising 12 case series and 14 case reports, with a total of 46 patients were included. Overall, the median (IQR) age of the pooled cohort was 18 (15–26) years, the median (IQR) time to presentation was 6 (3–36) hours from the onset of testicular pain. The most common presenting features were testicular pain (100%), testicular swelling (60.9%) and a high riding testicle (34.8%). The left testicle was most commonly affected (63.0%), RTT was on the ipsilateral side in relation to the primary episode of TT in 52.2% of cases, the median (IQR) interval between torsion and retorsion events was 4 (1.3–10.0) years, non‐absorbable sutures were the most common suture material used during orchidopexy after RTT (88.9%).

**Conclusion:**

RTT is a rare presentation to the Emergency Department. Even with a prior history of TT, RTT should be considered in patients presenting with classic symptoms.

## Graphical abstract

Recurrent testicular torsion following previous surgical exploration and management of testicular torsion is a rarely reported event. Previous testicular surgery does not guarantee permanent fixation of the testis, even after bilateral orchidopexy. Failure to consider the rare possibility of RTT may delay diagnosis and result in testicular loss.

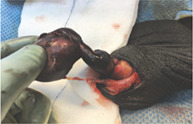

## Introduction

Testicular torsion (TT) is a urological emergency that commonly presents to the emergency department (ED). It occurs as a result of rotation of the testis around the spermatic cord, thereby compromising testicular blood flow and resulting in irreversible ischemic testicular damage.^1^, ^2^ Testicular torsion can occur at any age but usually occurs in young males, with a bimodal incidence in the paediatric population: during the first year of life, and between the ages of 13 and 16 years.^3^ Timeous surgical exploration is required to untwist the ischemic testicle and thereafter fixate it (orchidopexy) to prevent retorsion. Identification and management should ideally be performed within 4–6 h of symptoms onset to prevent testicular infarction. If treated within 6 h of onset of pain, there is a greater chance of saving the affected testicle, as 90–100% testicles will be saved.^4^ Anderson *et al*. found that 89% of testes operated on within 7–12 h were salvaged.^5^ Salvage rates decline rapidly with time with less than 10% salvageability when the duration of torsion is greater than 24 hours.^6^ Since the contralateral testis is also predisposed to torsion, it is generally also fixated during the same procedure.^7^

Recurrent testicular torsion (RTT) following previous surgical exploration and management of TT is a rarely reported event.^8^ Patients may present to the ED with acute testicular pain, nausea, vomiting, a high riding testicle, a history of prior TT and a history of prior scrotal surgery.^9^ When the diagnosis of RTT is suspected, urgent surgical re‐exploration should be considered to prevent potential testicular loss.^7^ Hence, a history of previous testicular exploration should not exclude RTT as a diagnosis.

Various techniques have been described to reduce the incidence of testicular retorsion.^9^ The surgical technique used during the initial exploration as well as the suture type used have been linked to the cause of orchidopexy failure.^8^ Various studies have reported that RTT is more common when absorbable sutures were used during initial orchidopexy, leaving the affected testis to lose its attachments to the scrotal wall.^7^ Moreover, complications following a second orchidopexy, such as testicular atrophy, infertility, and chronic pain have not been explored in the literature.

Overall, there is a paucity of data pertaining to the presentation, diagnosis, risk factors, management, and outcomes of RTT post orchidopexy for TT. Therefore, a comprehensive literature review was performed using the current body of literature.

## Methods

### Search strategy

This systematic review was registered on the PROSPERO database (CRD42021258997) prior to commencement of the search. A search strategy was conducted in October 2021 using the following databases: PubMed, Google Scholar, Embase, Scopus, Web of Science, Cochrane Database of Systematic Reviews, Global Index Medicus and Cumulative Index to Nursing and Allied Health Literature (CIANHL). The following search terms were used: ‘recurrent testicular torsion’ OR ‘spermatic cord torsion’ AND (‘torsion’ OR ‘failure’ OR ‘recurrent’ OR ‘re‐operation’ OR ‘treatment’). All citations retrieved from the various papers were analysed for additional relevant resources. The search was restricted to publications within the medical literature. No language restrictions were applied.

### Study selection

Studies included in the review met the following criteria: (i) the studies were clinical publications, (ii) limited to human studies and (iii) included full study text. All publications relating to the topic, including correspondence and letters to the editors were eligible for inclusion. Exclusion criteria included: (i) studies that were found as a result of keyword matching or tags but are obviously irrelevant to the study topic, (ii) full text articles not available and (iii) articles pertaining to the first occurrence of TT, rather than RTT.

### Review study definition of recurrent testicular torsion

For the purpose of this systematic review, recurrent testicular torsion was defined as a representation of testicular torsion following previous orchidopexy.

### Data extraction and methodological evaluation

The Preferred Reporting Items for Systematic Reviews and Meta‐analyses (PRISMA) guidelines were applied to guide the electronic search.^10^ Articles fitting the eligibility criteria were screened by two independent reviewers (MVW & LQ), based on the inclusion criteria described above, a descriptive narrative of each study was compiled by the reviewers. Conflicting entries, disagreements and differences were resolved by seeking opinions from a third reviewers (AA). The points of interest in each study were tabulated. These included the study origin, age range of the study population, sample size, time from symptom onset to presentation, duration between episodes of torsion, signs and symptoms of RTT, tool used to diagnose RTT, degree of torsion rotation, surgical techniques used, suture materials and number of sutures used, comments on previous orchidopexy, complications and recommendations.

### Data synthesis

The outcomes reported from this systematic review were summary data pertaining to the clinical features of RTT, the diagnostic pathway of RTT, the common surgical techniques used for surgical exploration of RTT, and an overview of complications. Given the anticipated heterogeneity in reported data across studies, a narrative synthesis was primarily utilized outlining the range of techniques described. Descriptive summary statistics included the median and interquartile range (IQR) of the reported values for baseline variables such as age, time to presentation, interval between torsion and retorsion, degree of torsion and number of sutures. Other data including surgical techniques were tabulated and described.

### Assessment of methodological quality of included articles

**Fig. 1:**
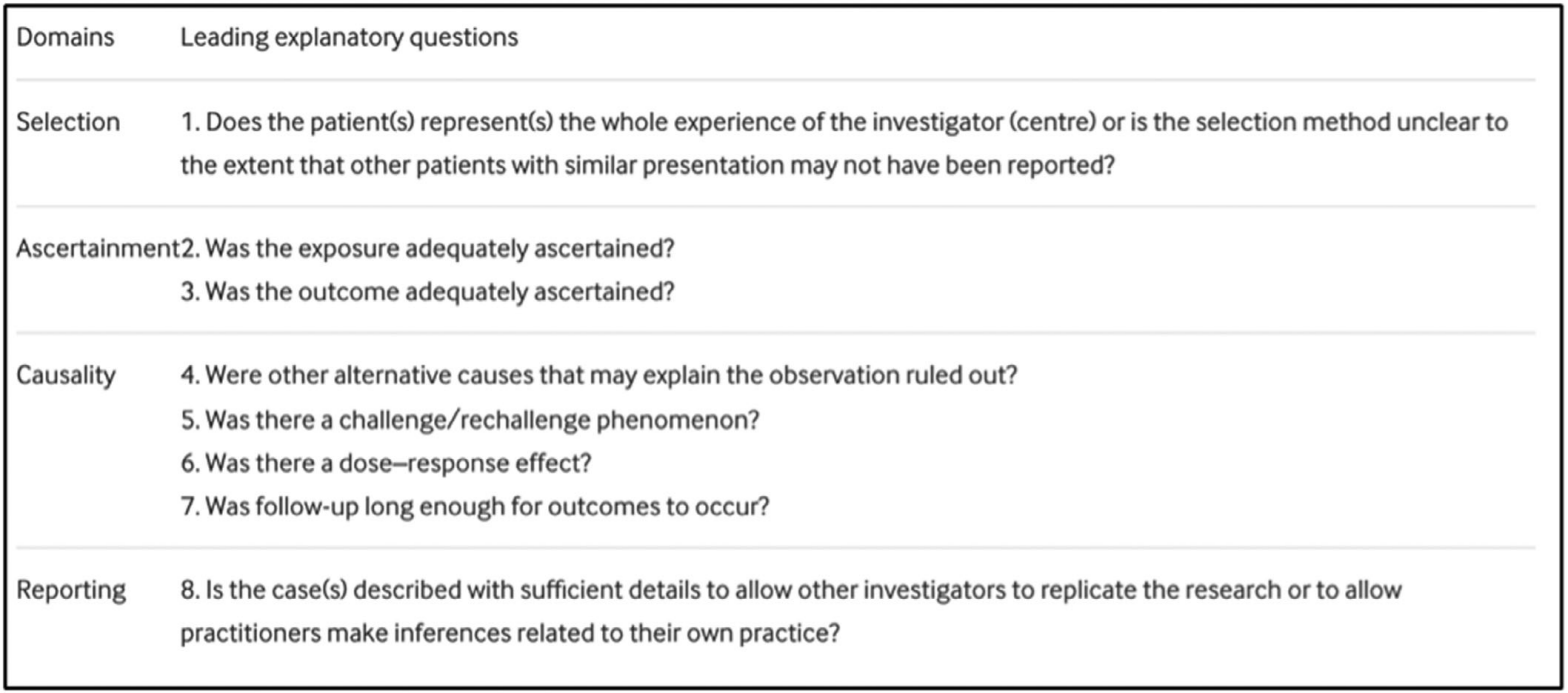
Tool for evaluating the methodological quality of case reports and case series (figure obtained from Murad MH, Sultan S, Haffar S, Bazerbachi F. methodological quality and synthesis of case series and case reports. Evid based med. 2018;23 (2):60–3. Doi: 10.1136/bmjebm‐2017‐110 853.^11^ Distributed under the terms of the creative commons attributions 4.0 international Licence (http://creativecommons.org/licenses/by‐nc/4.0/). No changes have been made to the figure or the figure description).

All studies that met the inclusion criteria were either case reports or case series. The methodological quality of the included articles were assessed using the tool proposed by Murad *et al*.^11^ The tool comprises four domains with a total of eight questions (Fig. 1). Since questions 5 and 6 were not relevant to our study as they predominantly relate to drug reactions, these were removed. The overall methodological quality of each of the included articles was described as either low quality, intermediate quality, or high quality. High quality was defined as a ‘yes’ answer to 4 or more of the included questions, while intermediate quality was defined as a ‘yes’ answer to 3 of the included questions and low quality was defined as a ‘yes’ answer to fewer than 3 of the included questions (Table 1).

**Table 1:** Methodological quality of included articles

First author	No. of cases	Q1	Q2	Q3	Q4	Q5	Q6	Overall Quality
Johenning^8^	2	Yes	No	Yes	No	Unclear	Yes	Intermediate
Kossow^12^	1	Unclear	No	No	No	Unclear	Yes	Low
May^13^	2	Yes	Yes	Yes	Yes	Unclear	Yes	High
McNellis^9^	4	Yes	Yes	Yes	Yes	Unclear	Yes	High
Redman^14^	1	Unclear	No	No	No	Unclear	Yes	Low
Vorstman^15^	1	Unclear	Yes	Yes	Yes	Unclear	Yes	High
Naughton^16^	1	Unclear	Yes	No	Yes	Unclear	Yes	Intermediate
Thurston^17^	5	Yes	No	Yes	No	Yes	Yes	High
Tawil^18^	1	Unclear	No	No	No	Unclear	Yes	Low
Kuntze^19^	2	Yes	Yes	Yes	Yes	Unclear	Yes	High
Gillion^11^	2	Yes	Yes	Yes	Yes	Unclear	Yes	High
Hulecki^20^	1	Unclear	Yes	No	Yes	Unclear	Yes	Intermediate
Morgan^21^	1	Unclear	Yes	No	Yes	Unclear	Yes	Intermediate
Phillips^22^	1	Unclear	Yes	No	Yes	Unclear	Yes	Intermediate
Steinbruchel^23^	2	Yes	No	No	No	Unclear	Yes	Low
O'Shaughnessy^24^	2	Yes	Yes	Yes	Yes	Unclear	Yes	High
Hurren^25^	2	Yes	Yes	No	Yes	Unclear	Yes	High
Chinegwundoh^26^	1	Unclear	Yes	No	Yes	Unclear	Yes	Intermediate
Rasmussen^27^	2	Yes	No	No	No	Unclear	No	Low
Von Zastrow^28^	4	Yes	Yes	No	Yes	Unclear	Yes	High
De Vylder^10^	3	Yes	Yes	No	Yes	Unclear	Yes	High
Blaut^29^	2	Yes	No	No	No	Unclear	No	Low
Van Glabeke^30^	1	Unclear	No	No	No	Unclear	Yes	Low
Alnadhari^31^	1	Unclear	No	No	No	Unclear	Yes	Low
Koochakzadeh^32^	1	Unclear	Yes	Yes	Yes	Unclear	Yes	High
Wang^33^	1	Unclear	Yes	No	Yes	Unclear	Yes	Intermediate

Abbreviation: Q, question.

*Note*: *Questions 1–6 comprise the tool for assessing the methodological quality of each of the included articles: Does the patient(s) represent(s) the whole experience of the investigator or is the selection method unclear to the extent that other patients with similar presentation may not have been reported? Was the condition adequately ascertained? Was the outcome adequately ascertained? Were other alternative causes that may explain the observation ruled out? Was follow‐up long enough for outcomes to occur? Is the case(s) described with sufficient details to allow other investigators to replicate the research or to allow practitioners to make inferences related to their own practice?

#### Outcomes

1. To describe the presentation of RTT
2. To describe the diagnosis RTT
3. To determine the risk factors associated with RTT
4. To describe the management and outcomes associated with RTT

## Results

### Search

**Fig. 2:**
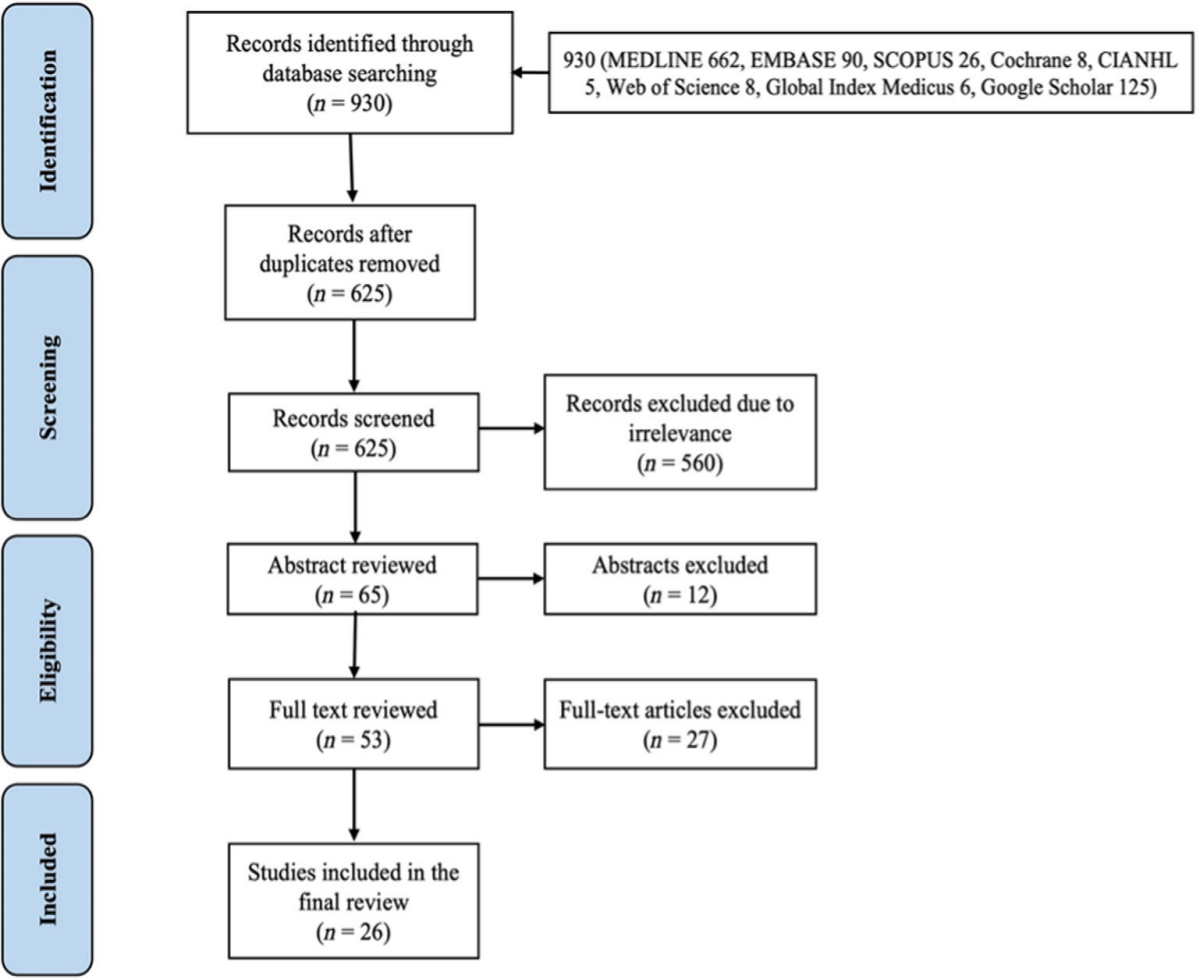
Study flow diagram.

The electronic database search yielded 930 titles with the following breakdown: PubMed (*n* = 662), Google Scholar (*n* = 125), Embase (*n* = 90), Scopus (*n* = 26), Web of Science (*n* = eight), Cochrane Database of Systematic Reviews (*n* = eight), Global Index Medicus (*n* = six) and CIANHL (*n* = five). Of these, 865 titles were excluded (305 duplicates and 560 irrelevant to the topic). A further 12 were removed after abstract review. The remaining 53 articles were fully reviewed, of which 26 articles were selected for inclusion in this systematic review. Details of the above are described in Figure 2.

### Study metadata

The 26 included articles^12^, ^13^, ^14^, ^15^, ^16^, ^17^, ^18^, ^19^, ^20^, ^21^, ^22^, ^23^, ^24^, ^25^, ^26^, ^27^, ^28^, ^29^, ^30^, ^31^, ^32^, ^33^, ^34^, ^35^, ^36^, ^37^ comprised 12 case series^12^, ^14^, ^15^, ^21^, ^23^, ^24^, ^27^, ^31^, ^34^, ^35^, ^36^, ^37^ and 14 case reports,^13^, ^16^, ^17^, ^18^, ^19^, ^20^, ^22^, ^25^, ^26^, ^28^, ^29^, ^30^, ^32^, ^33^ with a total of 46 patients with RTT. The largest of the case series included five subjects.^27^ The methodological quality of each of the included articles is described in Table 1. A total of 11 (42.3%) studies were ranked as high quality,^12^, ^14^, ^15^, ^17^, ^21^, ^23^, ^24^, ^26^, ^27^, ^36^, ^37^ seven (26.9%) as intermediate quality^13^, ^16^, ^20^, ^22^, ^29^, ^30^, ^31^ and 8 (30.1%) as low quality.^18^, ^19^, ^25^, ^28^, ^32^, ^33^, ^34^, ^35^ Details of the included articles are described in Tables 2, 3, 4.

**Table 2:** Summary of literature included in the review

	First author	Location of study	Year published	Sample size	Age (years)	Time from symptom onset to ED presentation (hours)	Time from initial surgical intervention to representation (years)
1	Johenning^8^	Ohio, USA	1973	2	17	36	14
					16	–	1
2	Kossow^12^	Florida, USA	1980	1	21	96	6
3	May^13^	Bristol, UK	1980	2	10	Few	2
					15	2	1
4	McNellis^9^	Pennsylvania, USA	1980	4	24	–	4
					16	–	0.58
					29	5	2
					16	–	0.75
5	Redman^14^	Arkansas, USA	1980	1	16	36	1.5
6	Vorstman^15^	Auckland, New Zealand	1982	1	15	Several	0.92
7	Naughton^16^	Dublin, Ireland	1983	1	16	8	4
8	Thurston^17^	Cambridge, UK	1983	5	26	48	11
					12	12	5
					28	120	16
					12	3	7
					15	3	0.83
9	Tawil^18^	Mussouri, USA	1984	1	23	48	5
10	Kuntze^19^	California, USA	1985	2	15	48	0.83
					17	16	4
11	Gillion^11^	Tel Aviv, Israel	1986	2	15	3	2
					12	Several	0.5
12	Hulecki^20^	Virginia, USA	1986	1	15	2	3
13	Morgan^21^	Texas, USA	1986	1	19	–	1
14	Phillips^22^	Leicester, UK	1987	1	12	4	8
15	Steinbruchel^23^	Kolding, Denmark	1988	2	26	24	15
					34	4	17
16	O'Shaughnessy^24^	Dublin, Ireland	1990	2	18	3	2
					20	6	6
17	Hurren^25^	Southhampton, UK	1992	2	20	Sudden	4
					33	6	27
18	Chinegwundoh^26^	Stoke‐on‐Trent	1995	1	20	4	6
19	Rasmussen^27^	Randers, Denmark	1996	4	20	6	11
					12	12	2
					26	4	7
					5	2	3.5
20	Von Zastrow^28^	Germany	2005	2	29	–	10
					16	–	1.17
21	De Vylder^10^	Netherlands	2006	3	22	–	4
					30	168	15
					35	–	–
22	Blaut^29^	Germany	2008	1	13	30	2
23	Van Glabeke^30^	France	2010	1	27	–	10
24	Alnadhari^31^	Doha, Qatar	2019	1	31	120	25
25	Koochakzadeh^32^	Florida, USA	2019	1	13	Sudden	1
26	Wang^33^	Ohio, USA	2019	1	22	3	14

**Table 3:** Summary of clinical features of cases included in the review

	First author	Abdominal pain	Nausea & vomiting	Testicular pain	Redness	Swelling	High riding testicle	Degree of rotation	Side of initial torsion	Side of recurrent torsion	How was diagnosis made?
1	Johenning^8^	–	–	X	X	X	‐	360	R	R	PE
		–	–	X	–	X	–	180	R	L	PE
2	Kossow^12^	–	X	X	–	X	–	180	R	L	PE
3	May^13^	–	–	X	–	X	–	–	–	–	PE
		–	X	X	X	X	–	–	R	R	Observations of Angell
4	McNellis^9^	–	–	X	–	–	X	–	L	R	PE
		–	–	X	–	–	–	–	R	R	Doppler & TS
		–	–	X	–	–	–	–	L	L	Doppler
		–	–	X	–	–	–	–	R	L	Surgery
5	Redman^14^	–	–	X	X	X	–	360	R	L	PE
6	Vorstman^15^	–	–	X	–	X	–	–	L	L	PE
7	Naughton^16^	–	–	X	–	X	X	–	L	L	PE
8	Thurston^17^	X	X	X	–	–	–	–	UD	L	PE
		–	–	X	–	X	X	–	RUD	R	PE
		–	–	X	X	X	X	540	LUD	R	PE
		–	–	X	–	X	–	–	R	L	PE
		–	–	X	–	–	–	–	L	L	PE
9	Tawil^18^	–	–	X	X	–	X	360	L	R	PE
10	Kuntze^19^	–	–	X	–	X	–	360	L	R	PE, Doppler, TS
		–	–	X	–	X	–	360	R	L	TS
11	Gillion^11^	–	–	X	–	–	–	–	R	L	PE
		–	–	X	–	–	–	180	R	L	PE
12	Hulecki^20^	–	–	X	–	–	X	720	R	L	Doppler & TS
13	Morgan^21^	–	X	X	–	–	X	–	L	L	PE & TS
14	Phillips^22^	–	–	X	X	X	X	720	RUD	R	PE
15	Steinbruchel^23^	–	X	X	–	X	–	–	LUD	L	PE
		–	–	X	–	X	X	720	RUD	R	PE
16	O′Shaughnessy^24^	–	–	X	–	–	–	540	RUD	L	PE
		–	–	X	–	X	X	360	LUD	R	PE
17	Hurren^25^	–	–	X	–	X	X	–	LUD	L	PE
		–	–	X	–	–	X	180	LUD	L	PE
18	Chinegwundoh^26^	–	–	X	–	X	–	–	R	L	Doppler
19	Rasmussen^27^	–	–	X	–	X	‐	–	RUD	L	PE
		–	–	X	–	X	‐	–	LUD	R	PE
		–	–	X	–	X	X	–	RUD	L	PE & Doppler
		–	–	X	–	X	X	360	RUD	L	PE
20	Von Zastrow^28^	–	–	X	–	–	–	–	R	R	Doppler
		–	–	X	X	X	–	1080	R	L	PE & Doppler
21	De Vylder^10^	–	–	X	–	–	–	720	L	L	PE
		–	–	X	X	–	–	–	R	R	PE
		–	–	X	–	X	–	–	R	L	Doppler
22	Blaut^29^	–	X	X	–	–	–	360	R	L	Doppler
23	Van Glabeke^30^	–	–	X	–	X	–	720	R	L	Doppler
24	Alnadhari^31^	–	–	X	–	X	–	–	L	L	Doppler
25	Koochakzadeh^32^	–	–	X	–	X	X	360	L	R	Doppler
26	Wang^33^	X	X	X	–	‐	X	360	L	L	Doppler

Abbreviations: L, left; LUD, left undescended; PE, physical exam; R, right; RUD, right undescended; TS, testicular scan; UD, undescended.

**Table 4:** Summary of surgical techniques for TT and RTT reported in the included articles

		First Incidence of Torsion					Second Incidence of Torsion				
	First author	Technique	Suture type	No. of sutures	Site of sutures	Complications	Technique	Suture type	No. of sutures	Site of sutures	Complications
1	Johenning^8^	–	–	–	–	None	TA to SW	–	2	–	R Orchiectomy
		TA to scrotum	3/0 chromic catgut	2	–	–	–	–	–	–	None
2	Kossow^12^	–	–	–	–	R Orchiectomy	Fixation	–	3	–	L Atrophy
3	May^13^	TA to PTV	Catgut	Several	–	None	PTV excised	–	–	–	None
		TA to PV	Catgut	3	–	None	PTV excised	–	–	–	R Atrophy
4	McNellis^9^	Fixed bilaterally	3/0 chromic catgut	–	–	None	Fixed bilaterally	–	–	–	None
		Fixed bilaterally	3/0 vicryl	–	–	–	Fixed bilaterally	–	–	–	–
		Fixed bilaterally	3/0 chromic catgut	–	–	–	Fixed bilaterally	–	–	–	–
		‐	Absorbable	–	–	–	Fixed bilaterally	Non‐absorbable	–	–	–
5	Redman^14^	PTV to VTV	3/0 chromic catgut	3	–	–	–	–	–	–	–
6	Vorstman^15^	TA to SS	3/0 chromic catgut	2	–	None	TA to SW	Silk	2	–	None
7	Naughton^16^	–	–	–	–	–	–	–	–	–	–
8	Thurston^17^	Fixed to thigh	Chromic catgut	1	–	–	Evag. TV to SW	Non‐absorbable	2	Bipolar	L Orchiectomy
		Dartos Pouch	–	–	–	None	Evagination PTV	Non‐absorbable	3	–	None
		–	–	–	Lower	L Orchiectomy	Plication of TV	–	–	–	–
		–	2/0 chromic catgut	–	Bipolar	R Orchiectomy	–	Non‐absorbable ‐	3	–	Swelling
		Fixed bilaterally	Absorbable	‐	Bipolar	None	–	–	–	–	None
9	Tawil^18^	VTV to TA & PTV	3/0 chromic catgut	2	–	–	–	–	–	–	R + L Orchiectomy
10	Kuntze^19^	Fixed to DF	2/0 chromic	–	–	L Orchiectomy	–	3/0 silk	‐	‐	R Orchiectomy
		TA to DF	3/0 chromic	–	–	R Orchiectomy	Remove window TV	3/0 silk	‐	‐	L Orchiectomy
11	Gillion^11^	Fixed bilaterally	–	–	–	–	–	Non‐absorbable	–	–	–
		Fixed bilaterally	–	–	–	–	Fixation to TV	Non‐absorbable	–	–	–
12	Hulecki^20^	Transeptally	2/0 proline	–	–	None	–	3/0 proline	4	–	None
13	Morgan^21^	–	–	1	–	–	–	2/0 vicryl	2	–	–
14	Phillips^22^	–	Catgut	2	Bipolar	–	–	Silk	3	–	–
15	Steinbruchel^23^	–	–	1	SW&TE	–	–	–	–	–	–
		–	Catgut	2	Lower	–	Fixed to DF	–	–	–	–
16	O′Shaughnessy^24^	DP to DF	–	–	–	–	TA to TV	2/0 polyglycolic acid	2	–	–
		DP fixation	Chromic catgut	1	–	–	–	2/0 polyglycolic acid	–	–	–
17	Hurren^25^	–	–	–	–	–	Fixed bilaterally	–	–	–	–
		–	–	–	–	–	Fixed bilaterally	–	–	–	–
18	Chinegwundoh^26^	–	Catgut	–	–	None	–	–	–	–	None
19	Rasmussen^27^	–	–	–	–	–	Fixed to DF	–	–	–	–
		–	–	–	–	–	Fixed to DF	–	–	–	–
		–	–	–	–	–	Fixed to DF	–	–	–	–
		–	–	–	–	–	Fixed to DF	Dexon	–	–	–
20	Von Zastrow^28^	DF to TA	Non‐absorbable	3	–	–	–	–	–	–	–
		–	–	–	–	–	R Orchiectomy	–	–	–	L Atrophy
21	De Vylder^10^	–	Absorbable	2	–	–	Jaboulay's bottle neck	–	–	–	–
		Fixed bilaterally	Absorbable	–	–	–	–	–	–	–	R Orchiectomy
		Fixed to TA	–	–	–	R Orchiectomy	–	–	–	–	–
22	Blaut^29^	–	–	–	–	–	R Orchiectomy	4/0 polypropylene	3	SW	L Orchiectomy
23	Van Glabeke^30^	–	–	–	–	–	R Orchiectomy	–	–	–	L Orchiectomy
24	Alnadhari^31^	–	–	–	Lower	–	Fixed to SW	–	3	–	L Orchiectomy
25	Koochakzadeh^32^	Fixation to DP	4/0 prolene	4	–	L Orchiectomy	TA to SS	4/0 non‐absorbable	4	–	–
26	Wang^33^	–	5/0 prolene	3	Lower pole	–	–	4/0 prolene	3	–	–

Abbreviations: DF, dartos fascia; DP, dartos pouch; PTV, parietal tunica vaginalis; SS, scrotal septum; SW, scrotal wall; TA, tunica albuiginea; TE, tail of epididymis; VTV, visceral tunica vaginalis.

### Definition of RTT among the included studies

In general, the included articles all alluded to the definition of RTT as a recurrence of testicular torsion following previous orchidopexy.

### Region of publication of included articles

Among the included articles, 10 (38.5%) emanated from the USA,^15^, ^16^, ^19^, ^20^, ^21^, ^22^, ^26^, ^31^, ^32^, ^33^ five (19.2%) from the UK,^23^, ^24^, ^27^, ^29^, ^30^ two (7.7%) each from Ireland,^13^, ^37^ Denmark,^35^, ^36^ and Germany^28^, ^34^ and one (3.8%) each from New Zealand,^17^ Israel,^12^ the Netherlands,^14^ France^25^ and Qatar.^18^

### Age range of study subjects

The age range of all included subjects ranged from 5–35 years. The median (IQR) age of the pooled cohort was 18 (15–26) years. Approximately three‐quarter of the pooled subjects (*n* = 34, 73.9%) were younger than 25 years of age.^12^, ^13^, ^14^, ^15^, ^16^, ^17^, ^19^, ^20^, ^21^, ^22^, ^23^, ^24^, ^27^, ^28^, ^29^, ^30^, ^31^, ^32^, ^33^, ^34^

### Time to ED presentation from presumed onset of RTT

Among the 36 (78.3%) cases in whom time to ED presentation from the presumed onset of RTT was reported, the median (IQR) time was 6 (3–36) hours after the onset of testicular pain.^12^, ^13^, ^14^, ^15^, ^16^, ^17^, ^18^, ^19^, ^21^, ^22^, ^23^, ^24^, ^25^, ^26^, ^27^, ^28^, ^29^, ^30^, ^31^, ^32^, ^33^, ^34^, ^35^, ^36^, ^37^

### Presenting features of RTT

The most common signs and symptoms on RTT were testicular pain (*n* = 46, 100%),^12^, ^13^, ^14^, ^15^, ^16^, ^17^, ^18^, ^19^, ^20^, ^21^, ^22^, ^23^, ^24^, ^25^, ^26^, ^27^, ^28^, ^29^, ^30^, ^31^, ^32^, ^33^, ^34^, ^35^, ^36^, ^37^ testicular swelling (*n* = 28, 60.9%),^13^, ^14^, ^15^, ^18^, ^19^, ^23^, ^24^, ^26^, ^27^, ^29^, ^30^, ^31^, ^32^, ^34^, ^37^ a riding high testicle (*n* = 16, 34.8%),^13^, ^16^, ^19^, ^20^, ^21^, ^22^, ^24^, ^26^, ^27^, ^30^, ^35^, ^36^, ^37^ testicular redness (*n* = 8, 17.4%),^14^, ^19^, ^23^, ^27^, ^30^, ^31^, ^32^, ^34^ nausea and/or vomiting (*n* = 7, 15.2%)^16^, ^20^, ^23^, ^27^, ^28^, ^33^, ^35^ and abdominal pain (*n* = 2, 4.4%).^16^, ^27^

### Affected side of TT and RTT

At the initial presentation of TT, the right testicle was more commonly affected (n = 26, 56.5%),^12^, ^14^, ^15^, ^21^, ^22^, ^25^, ^27^, ^28^, ^29^, ^31^, ^32^, ^33^, ^34^ whereas at the recurrent presentation, the left testicle was more commonly affected (*n* = 29, 63.0%).^12^, ^13^, ^14^, ^15^, ^16^, ^17^, ^18^, ^20^, ^21^, ^22^, ^24^, ^25^, ^27^, ^28^, ^29^, ^31^, ^32^, ^33^, ^34^, ^35^, ^36^ Overall, RTT was on the ipsilateral side in 24 (52.2%) cases ^13^, ^14^, ^16^, ^17^, ^18^, ^20^, ^21^, ^23^, ^24^, ^27^, ^30^, ^31^, ^34^, ^35^, ^37^ and on the contralateral side in 22 (47.8%) cases.^14^, ^15^, ^19^, ^21^, ^22^, ^25^, ^26^, ^27^, ^28^, ^29^, ^31^, ^32^, ^33^, ^34^, ^36^, ^37^

### Interval between initial TT and RTT episodes

Among the 45 (97.8%) cases^12^, ^13^, ^14^, ^15^, ^16^, ^17^, ^18^, ^19^, ^20^, ^21^, ^22^, ^23^, ^24^, ^25^, ^26^, ^27^, ^28^, ^29^, ^30^, ^31^, ^32^, ^33^, ^34^, ^35^, ^36^, ^37^ in whom the interval between torsion and retorsion episodes was reported, the median (IQR) interval was 4 (1.3–10.0) years, with the shortest duration being 6 months^12^ and the longest 27 years.^24^

### Degree of torsion at RTT episode

Among the 26 (56.5%) cases^12^, ^14^, ^15^, ^16^, ^19^, ^22^, ^24^, ^25^, ^26^, ^27^, ^28^, ^30^, ^31^, ^32^, ^33^, ^34^, ^35^, ^36^, ^37^ in whom the rotational degree of RTT was reported, the median (IQR) degree of rotation of the testis was 360 (360–720)°, with the largest degree of torsion being 1080° (i.e., a twist torsion of three rotations).^34^

### Diagnostic modality utilized to diagnose RTT

Physical examination was most frequently relied upon as the sole means of diagnosing RTT in 32 (69.5%)^12^, ^13^, ^14^, ^15^, ^17^, ^19^, ^20^, ^21^, ^23^, ^24^, ^27^, ^30^, ^31^, ^32^, ^33^, ^34^, ^35^, ^36^, ^37^ cases. Doppler ultrasound was performed in 13 (28%) cases,^14^, ^15^, ^16^, ^18^, ^21^, ^22^, ^25^, ^26^, ^28^, ^29^, ^34^, ^36^ while a testicular nuclear scan was performed in five (10.8%) cases.^15^, ^20^, ^21^, ^22^ Eleven (23.9%) cases were misdiagnosed, of which seven (15.2%) were misdiagnosed as epididymitis^18^, ^19^, ^25^, ^31^, ^33^, ^34^, ^35^ and one (2.2%) as spermatic cord neuralgia.^34^

### Surgical technique utilized for TT and RTT

The surgical techniques used during the index episode of TT was only described in 24 (52.8%) cases. Of these, in eight (17.4%) cases, the authors simply reported that the testes were fixed bilaterally,^12^, ^14^, ^21^, ^22^, ^27^ while a dartos pouch fixation (either to the tunica albuginea or tunica vaginalis) was described in nine (19.6%) cases,^15^, ^19^, ^26^, ^27^, ^34^, ^37^ and the tunica albuginea was described to be fixed either to the parietal tunica vaginalis (*n* = 2, 4.4%),^35^ visceral tunica vaginalis (*n* = 1, 2.2%)^14^ or to the scrotum itself (*n* = 2, 4.4%) in another five cases.^17^, ^31^ Two other authors describe unusual techniques for fixation as fixed to the thigh (*n* = 1, 2.2%)^27^ and pexed transeptally (*n* = 1, 2.2%).^22^

The surgical techniques used during RTT was only described in 26 cases (56.5%). In seven (15.2%) cases, the authors simply reported that the testes were fixed bilaterally.^21^, ^24^, ^37^ In another seven cases it was described as fixation of the testes to the dartos (*n* = 5, 10.9%),^35^, ^36^ scrotal wall (*n* = 1, 2.2%)^18^ and tunica vaginalis (*n* = 1, 2.2%),^27^ in four cases it was described as fixation of the tunica albuginea to the scrotal wall (*n* = 2, 4.4%),^17^, ^31^ the septum (*n* = 1, 2.2%)^26^ and the tunica vaginalis (*n* = 1, 2.2%),^37^ while in eight (17.4%) cases,^14^, ^23^, ^27^ Jaboulay's technique (external eversion of the tunica vaginalis with suturing of the free edges posterior to the spermatic cord)^38^ was used.

### Type and number of sutures used

Of the 25 cases in which the suture type for initial presentation of TT was reported, absorbable sutures were used in 21 (84.0%) cases, with a median (IQR) of 2 (1–3) sutures used per case.^14^, ^15^, ^17^, ^19^, ^21^, ^23^, ^27^, ^29^, ^30^, ^31^, ^32^, ^35^, ^37^ Of the 18 cases in which the suture type for RTT was reported, non‐absorbable sutures were used in 16 (88.9%) cases with a median (IQR) of 3 (2, 3) sutures used per case.^12^, ^15^, ^16^, ^17^, ^21^, ^22^, ^26^, ^27^, ^28^, ^30^ Overall, the most widely used absorbable suture was chromic catgut (*n* = 11, 23.9%).^15^, ^17^, ^19^, ^21^, ^27^, ^31^, ^32^, ^37^ None of the studies specified the suture type for non‐absorbable sutures.

### Complications post RTT

In 20 (43.5%) cases, no mention was made as to the presence or absence of any complications.^12^, ^13^, ^14^, ^16^, ^20^, ^21^, ^24^, ^26^, ^27^, ^30^, ^32^, ^34^, ^35^, ^36^, ^37^ In a further 12 (26.1%) cases, the authors reported that there were no complications.^17^, ^22^, ^23^, ^27^, ^29^, ^31^ Among the 14 (30.4%) cases where complications were reported, 10 (21.8%) underwent orchidectomy,^14^, ^15^, ^18^, ^19^, ^25^, ^27^, ^28^, ^31^ three (6.5%) developed testicular atrophy^23^, ^33^, ^34^ and one (2.2%) had prolonged testicular swelling.^27^ Only one (2.2%) study reported low sperm count in a patient that underwent orchidectomy post RTT.^27^

### Recommendations

**Fig. 3:**
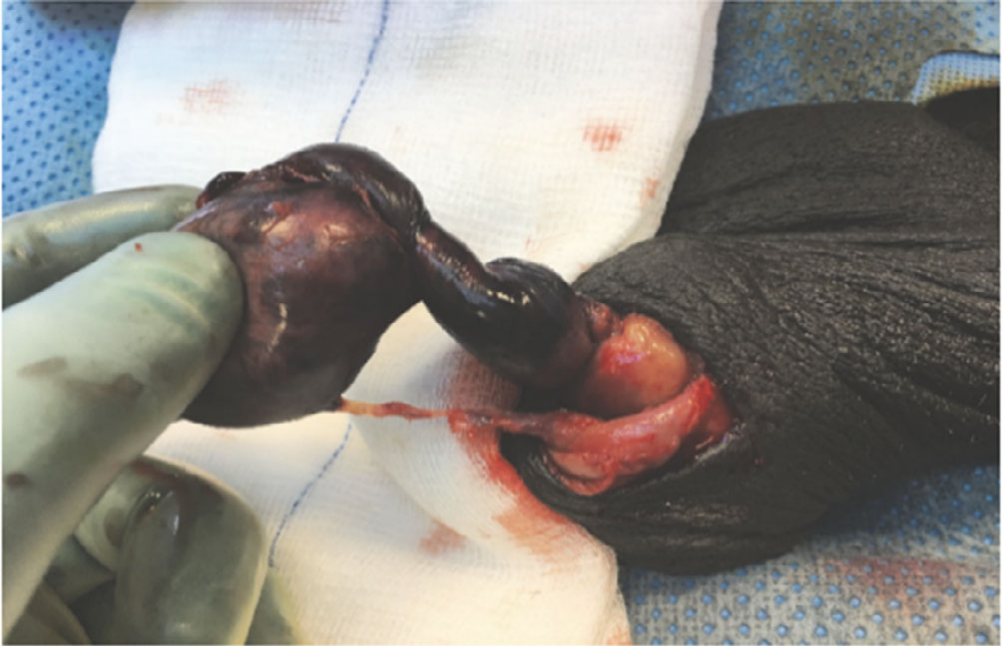
Black, torsed left testis attached with old stitch from lower pole to the side wall of scrotum (image obtained from Alnadhari I, Abdulmuhsin A, Ali O, Shamsodini A, Salah M, Abdeljaleel O. Recurrent testicular torsion of a fixed testis. Case rep Urol. 2019 Jul 15;1–3. Doi: 10.1155/2019/8735842.^18^ Distributed under the terms of the creative commons attributions 4.0 international Licence (http://creativecommons.org/licenses/by‐nc/4.0/). No changes have been made to the image or the image description).

Twenty‐four of the 26 (92.3%) authors included in this review concurred that the preferred method of treatment was to concurrently fixate both testes on initial presentation of TT.^12^, ^13^, ^14^, ^15^, ^16^, ^17^, ^18^, ^19^, ^20^, ^21^, ^22^, ^23^, ^24^, ^25^, ^26^, ^27^, ^29^, ^30^, ^31^, ^32^, ^33^, ^35^, ^36^, ^37^ The use of non‐absorbable sutures for fixation of initial TT was recommended by 14 (53.8%) authors,^12^, ^15^, ^16^, ^17^, ^18^, ^19^, ^21^, ^24^, ^25^, ^26^, ^27^, ^30^, ^32^, ^35^ while four (8.7%) authors recommended the use of three sutures sites^16^, ^19^, ^27^, ^29^ and six (23.1%) authors recommended Jaboulay's procedure (Fig. 3).^12^, ^14^, ^24^, ^26^, ^27^, ^31^

## Discussion

The incidence of RTT is probably higher than the literature would indicate.^39^ Previous testicular surgery does not guarantee permanent fixation of the testis, even after bilateral orchidopexy.^35^ Failure to consider the rare possibility of RTT may delay the diagnosis and result in testicular loss.^26^

The clinical diagnosis of RTT can be difficult due to the presence of non‐specific clinical signs and symptoms. Common symptoms, as shown in this review, include testicular pain, swelling, redness, nausea, vomiting and a high‐riding testicle. These symptoms are almost identical to TT.^40^ Patients who present with these symptoms were mostly younger than 25 years of age. This systematic review indicates that right testicle was more commonly involved during the initial presentation (56.5%), while the left testicle was more commonly involved in the recurrent episode (63.0%) and all patients had bilateral fixation at initial presentation of TT as well as at the recurrent episode. The bell‐clapper deformity is often found in patients who present with RTT. It is thought that patients who have the bell‐clapper deformity are at higher risk of torsion as there is increased mobility of the testicle within the tunica vaginalis.^6^

RTT presents with similar symptoms to TT, which can confuse and delay the diagnosis. If symptoms are equivocal, colour Doppler Ultrasound (US) has been shown to assist with confirming the diagnosis.^41^ The use of Doppler US is the favoured diagnostic modality to assess testicular blood flow, as it provides a non‐invasive view of the testes, is generally available at the bedside and can be performed rapidly.^42^ The use of US has a diagnostic accuracy of 95% in testicular torsion, similar to that achieved with radionuclear testicular scanning.^43^ When considering the diagnosis of RTT, physical exam findings are just as important to assess as they effectively guide diagnosis.^44^ The most definitive way to diagnose RTT is through surgical exploration. Surgical exploration should not be delayed, as this may lead to worsening ischemia and potential testicular loss.^1^, ^39^

Techniques to fixate the testes after torsion has shifted over the years. Suture types have changed and gained and lost favour among physicians. It was initially thought that absorbable sutures would be better, as it was assumed that a dense inflammatory response would limit rotational movement of the testis once the sutures had dissolved.^8^ However, various studies note that only fine adhesions form at the suture site, allowing potential retorsion to occur.^17^, ^21^, ^23^, ^27^, ^31^, ^32^ The widespread use of absorbable sutures in initial TT may also be related to standard recommendations in urological educational resources.^7^ It was only in 1992 that the recommended suture type was changed to non‐absorbable sutures. Within this review, only one case was reported using absorbable sutures after 1992.^14^ Many authors have advocated for the use of non‐absorbable sutures in the fixation of the testes. Among the reported cases, 89% used non‐absorbable sutures.^12^, ^15^, ^16^, ^17^, ^21^, ^22^, ^26^, ^27^, ^28^, ^30^ The shift in suture type used, however, did not prevent retorsion. Despite the use of non‐absorbable sutures at the initial presentation of TT, the occurrence of RTT was still reported.^16^, ^22^, ^26^, ^34^ The mechanism for torsion is therefore unclear, with little consensus among authors.

Surgical fixation techniques have also changed over the years. The tunica albuginea can be fixed to the scrotal wall, septum or tunica vaginalis.^17^, ^26^, ^31^, ^37^ A recent systematic review looked at different surgical techniques for orchidopexy.^39^ In this study, the authors attempted to find consensus among the various proposed techniques based on the available literature. There were several techniques presented, and it was reported that regardless of the technique used, there was no report of retortion in follow‐up at 6–31 weeks. This may suggest that all techniques were effective in the short term. It was noted, however, that there was a large degree of heterogeneity, high risk of bias and poor reporting of outcomes in the included studies. Moore *et al*. advise the need for an interim consensus until a randomized control trial can be conducted to determine the safest technique.^39^

Most authors agreed that both testes should be fixed,^12^, ^13^, ^14^, ^15^, ^16^, ^17^, ^18^, ^19^, ^20^, ^21^, ^22^, ^23^, ^24^, ^25^, ^26^, ^27^, ^29^, ^30^, ^31^, ^32^, ^33^, ^35^, ^36^, ^37^ regardless of the previous diagnosis. The use of non‐absorbable sutures, at three sites is recommended.^16^, ^19^, ^27^, ^29^ The fixation of the tunica albuginea to the dartos muscle, as well as eversion of the tunica albuginea, has been shown, at least in the current available literature, to prevent retorsion.^45^ It must be noted, however, that there is currently no available literature on the third occurrence of torsion, which may be a consequence not yet reported. Current surgical technique recommendations include orchidopexy of both testes with non‐absorbable sutures with fixing of the testes via the dartos pouch or directly to the dartos pouch.^9^ Based on animal data, it is recommended to perform a dartos pouch placement to preserve fertility.^9^

The reporting of complications may be challenging with few included studies having looked at long term outcomes. The link between delayed diagnosis and poor testicular outcome has been briefly commented upon. Often, patients are misdiagnosed as having epididymitis with physicians dismissing the possibility of recurrent torsion due to previous orchidopexy, which may lead to potential delays in diagnosis, testicular loss and subsequent litigation.^46^ Anderson *et al*. found that 89% of testes that underwent surgical intervention within 7–12 h were salvaged and that these rates rapidly declined over time.^5^ Less than 10% of testes were salvaged if the duration of torsion was greater than 24 h. None of the case studies in this review that reported testicular pain of longer than 24 h duration had testicular salvage.

The quality of the included studies was overall rated as low. This is due to all the included studies being case reports and case studies. This it in itself is a limitation as no multi‐centre studies exist to determine accurate information regarding RTT. This is probably due to the fact that the incidence of RTT is incredibly rare, and the earliest description of this event takes place in the 1970s.^31^ There is a need for larger studies to be done to further describe this condition.

## Conclusion

The diagnosis of RTT is complicated by the rarity of the condition and undifferentiated presentation. A high index of suspicion is required in the detection of this surgical emergency, as cases have been reported to occur even as late as two decades after the primary TT repair. There is little consensus regarding the optimal fixation technique. Absolute predictors for RTT were not identified within this review. Future research is required to further characterize this uncommon emergency.

## Conflicts of interest

None declared.

## Author contributions

**Mikayla van Welie:** Formal analysis; investigation; methodology; resources; writing – original draft. **Liang Qu:** Conceptualization; supervision; writing – review and editing. **Ahmed Adam:** Conceptualization; supervision; writing – review and editing. **Nathan Lawrentschuk:** Conceptualization; supervision; writing – review and editing. **Abdullah E Laher:** Conceptualization; project administration; supervision; writing – review and editing.
